# Proteomic Analysis of Mesenchymal Stromal Cells Secretome in Comparison to Leukocyte- and Platelet-Rich Fibrin

**DOI:** 10.3390/ijms241713057

**Published:** 2023-08-22

**Authors:** Niyaz Al-Sharabi, Reinhard Gruber, Mariano Sanz, Samih Mohamed-Ahmed, Einar K. Kristoffersen, Kamal Mustafa, Siddharth Shanbhag

**Affiliations:** 1 Center for Translational Oral Research (TOR), Department of Clinical Dentistry, Faculty of Medicine, University of Bergen, 5009 Bergen, Norway; n.al-sharabi@uib.no (N.A.-S.); samih.ahmed@uib.no (S.M.-A.); kamal.mustafa@uib.no (K.M.); 2 Department of Oral Biology, University Clinic of Dentistry, Medical University of Vienna, 1090 Vienna, Austria; reinhard.gruber@meduniwien.ac.at; 3 Austrian Cluster for Tissue Regeneration, 1090 Vienna, Austria; 4 Department of Periodontology, School of Dental Medicine, University of Bern, 3012 Bern, Switzerland; 5 ETEP Research Group, Faculty of Odontology, University Complutense of Madrid, 28040 Madrid, Spain; marsan@ucm.es; 6 Department of Immunology and Transfusion Medicine, Haukeland University Hospital, 5021 Bergen, Norway; einar.kleboe.kristoffersen@helse-bergen.no; 7 Department of Clinical Science, Faculty of Medicine, University of Bergen, 5021 Bergen, Norway

**Keywords:** mesenchymal stem cells, cell conditioned media, leukocyte- and platelet-rich fibrin, tissue engineering, regenerative medicine

## Abstract

Secretomes of mesenchymal stromal cells (MSCs) are emerging as a novel growth factor (GF)-based strategy for periodontal and bone regeneration. The objective of this study was to compare the secretome of human bone marrow MSC (BMSC) to that of leukocyte- and platelet-rich fibrin (L-PRF), an established GF-based therapy, in the context of wound healing and regeneration. Conditioned media from human BMSCs (BMSC-CM) and L-PRF (LPRF-CM) were subjected to quantitative proteomic analysis using liquid chromatography with tandem mass spectrometry. Global profiles, gene ontology (GO) categories, differentially expressed proteins (DEPs), and gene set enrichment (GSEA) were identified using bioinformatic methods. Concentrations of selected proteins were determined using a multiplex immunoassay. Among the proteins identified in BMSC-CM (2157 proteins) and LPRF-CM (1420 proteins), 1283 proteins were common. GO analysis revealed similarities between the groups in terms of biological processes (cellular organization, protein metabolism) and molecular functions (cellular/protein-binding). Notably, more DEPs were identified in BMSC-CM (*n* = 550) compared to LPRF-CM (*n* = 118); these included several key GF, cytokines, and extracellular matrix (ECM) proteins involved in wound healing. GSEA revealed enrichment of ECM (especially bone ECM)-related processes in BMSC-CM and immune-related processes in LPRF-CM. Similar trends for intergroup differences in protein detection were observed in the multiplex analysis. Thus, the secretome of BMSC is enriched for proteins/processes relevant for periodontal and bone regeneration. The in vivo efficacy of this therapy should be evaluated in future studies.

## 1. Introduction

Regeneration of advanced bone and periodontal defects is a clinical challenge [1,2]. Regeneration is the physiological renewal of tissue based on the principles of wound healing—the physiological response to injury [3,4]. Wound healing is a dynamic and well-orchestrated process consisting of four continuous, temporally overlapping phases, involving cellular and molecular events that occur after the onset of injury to restore the damaged tissue: hemostasis, inflammation, proliferation/angiogenesis, and extracellular matrix (ECM) (re)modeling [5,6].

Tissue engineering aims to facilitate or enhance the wound healing process via delivery of extrinsic factors in the form of biomaterials (autologous, allogeneic, or alloplastic), cells (autologous or allogeneic), and/or bioactive molecules (autologous, allogeneic, or recombinant). Among the most commonly applied tissue engineering strategies today are growth factors (GFs) [7] and adult mesenchymal stem/stromal cells (MSCs) [8]. The former mainly involves the use of recombinant human GFs or tissue fractions containing autologous GFs, such as autologous platelet concentrates (APCs) of the first-generation (platelet-rich plasma (PRP)) and second-generation (platelet-rich fibrin (PRF)) [9,10]. Recently, leukocyte- and platelet-rich fibrin (L-PRF) has received widespread interest due to its relative ease of preparation and reportedly high GF content [11,12,13]. L-PRF matrices can be prepared via “chair-side” centrifugation of whole blood without any additives or anticoagulants, resulting in a fibrin mesh with entrapped platelets, leucocytes, monocytes, and progenitor cells [14,15]. The secretome of L-PRF matrices also contains a complex mixture of GFs and other bioactive molecules which drive wound healing [16,17,18,19]. Moreover, we [16] and others [20] have previously identified potential pathways of L-PRF bioactivity based on in vitro assays using human fibroblasts and mesenchymal cells. The biological activity of L-PRF and its conditioned media (LPRF-CM) forms the basis for its clinical use in bone and periodontal tissue regeneration [21,22,23].

Cell therapy currently represents the state-of-the-art in tissue engineering and regenerative medicine [24]. Adult MSCs, usually from the bone marrow (BMSCs), are combined with GFs and/or biomaterial scaffolds to replicate the properties of autologous tissue grafts [25,26]. However, the need for expensive ex vivo laboratories and stringent regulation by health authorities has limited the widespread application of cell therapies [24]. Although the original rationale for cell therapy was based on the self-renewal and differentiation of MSC, recent preclinical data suggest alternative mechanisms of MSC bioactivity based on paracrine secretions and immune modulation instead of engraftment and direct replacement of injured tissues [27,28]. These rationales are based on MSC “secretomes”, i.e., a wide range of bioactive factors including soluble proteins (GFs, cytokines, chemokines), lipids, nucleic acids, and extracellular vesicles (EVs) [28,29,30]. These factors are reported to exert anti-inflammatory, anti-scarring, and immunomodulatory effects in vivo [31]. Additionally, our group has recently reported antiapoptotic effects of MSC secretomes [32]. These data provide the biological basis for utilizing the conditioned media, i.e., the culture media containing biologically active components secreted by BMSCs (BMSC-CM), in so-called “cell-free” tissue engineering strategies for regeneration [33,34,35]. Promising results have been reported following preclinical applications of BMSC-CM for bone and periodontal regeneration [34,35,36].

The efficacy of GF-based therapies depends largely on their proteomic composition. Given the frequent ex vivo characterization and widespread clinical use of L-PRF, it represents a standard to which newer GF-based strategies, such as MSC secretomes, may be compared, and which, to our knowledge, has not yet been reported. Therefore, the objective of the present study was to compare the secretome of BMSCs (BMSC-CM) to that of L-PRF (LPRF-CM) in terms of their proteomic profiles in the context of wound healing and regeneration.

## 2. Results

### 2.1. Proteomic Profiles of BMSC-CM and LPRF-CM

**Figure 1:**
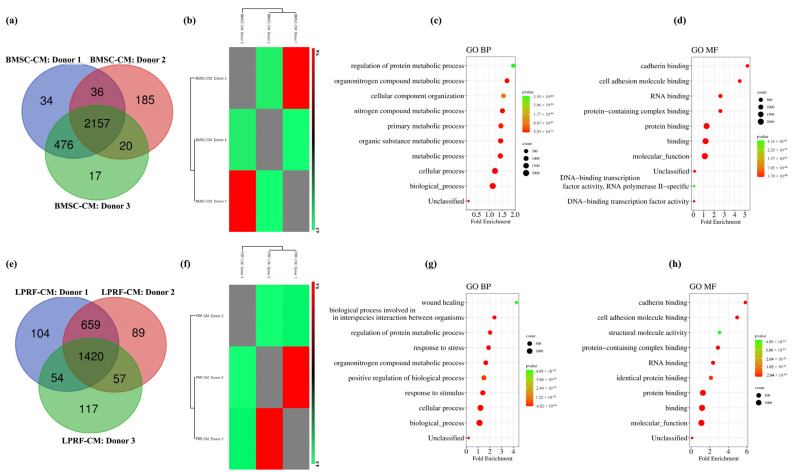
Assessment of the BMSC-CM and LPRF-CM proteomes. Venn diagrams showing distribution of CM proteins among the three BMSC donors (**a**) and three L-PRF donors (**e**). Protein intensity correlation analysis based on all three samples of BMSC-CM (**b**) and LPRF-CM (**f**). GO annotations and the top 10 annotated terms reflecting biological processes (BPs) and molecular functions (MFs) in BMSC-CM (**c**,**d**) and LPRF-CM (**g**,**h**). The enriched GO terms (BP, MF) are retrieved from the Gene Ontology resource and presented as enrichment bubble plot using SRplot; left *Y*-axis represents GO terms and *X*-axis represents fold enrichment; right *Y*-axis indicates protein counts (*n*) and *p*-values ranging from green (large, lower significance) to red (small, higher significance).

Proteomic analysis revealed 2952 proteins in BMSC-CM and 2500 proteins in LPRF-CM (Supplementary File S1). To ensure accurate quantification of proteins, two filtration strategies were applied based on detection of proteins in (a) at least two donors or (b) in all three donors, in each CM group. Based on the latter criterion, a total of 2157 and 1420 shared CM proteins were detected among the BMSC and L-PRF donors, respectively (Figure 1a,e). Protein intensity correlation analysis showed strong correlations between donors within each CM group, with average Pearson R values of 0.89 (range 0.85–0.98) for BMSC-CM and 0.85 (range 0.80–0.95) for LPRF-CM (Figure 1b,f).

GO analysis revealed similarities between BMSC-CM and LPRF-CM. BMSC-CM proteins were specifically associated with two main BPs: regulation of cellular components/functions and protein metabolic processes (Figure 1c), while the associated MF categories were related to binding functions (Figure 1d). Similarly, LPRF-CM proteins were also mainly linked to the organization and regulation of cellular components/functions and metabolic processes; additionally, “response to stress/stimulus” and “wound healing” processes were identified (Figure 1g). Analysis of MF in LPRF-CM also revealed associations with binding functions (Figure 1h).

### 2.2. Similarities and Differences between BMSC and L-PRF Secretomes

**Figure 2:**
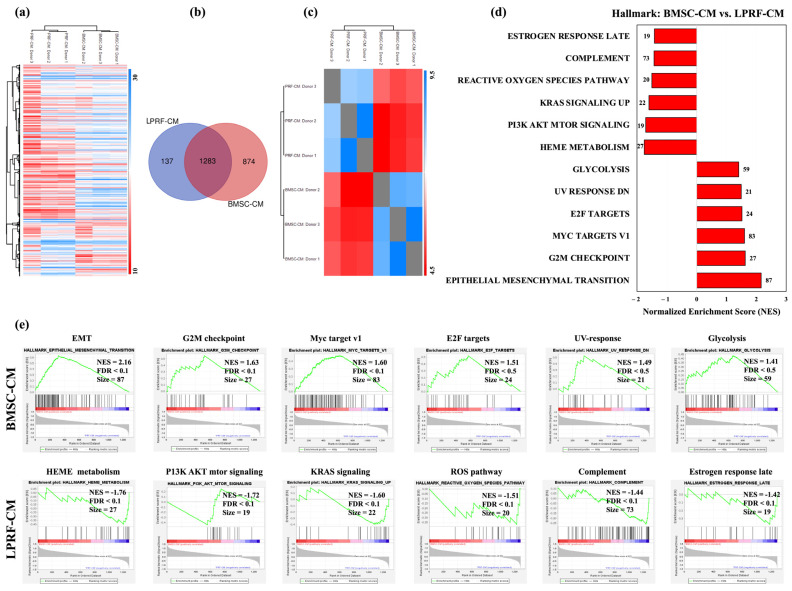
Gene set enrichment analysis (GSEA) of BMSC-CM and LPRF-CM. (**a**) Hierarchical clustering analysis of the shared proteins for each CM group in the comparison of LPRF-CM donors (right column) and BMSC-CM donors (left column); expression values are represented as colors and range from blue (high expression), white (moderate), to red (low expression). (**b**) Venn diagram illustrating the number of differentially expressed proteins (DEPs) in each CM group. (**c**) Protein intensity correlation analysis based on all samples showing detectable intensity levels for all three donors in each CM group: Pearson R ≥ 0.4 suggesting weak correlation between the BMSC-CM and LPRF-CM populations. (**d**) Results of GSE/hallmark analysis showing significantly enriched gene sets in each group (FDR < 25%); a positive normalized enrichment score (NES) indicates enrichment in the BMSC-CM group, while a negative NES indicates enrichment in the LPRF-CM group. (**e**) Enrichment plots for all significant datasets enriched in GSE/hallmark analysis at FDR < 25% showing the profile of the NES, FDR ratio, and number of proteins.

Hierarchical clustering revealed differences in the global profiles of BMSC-CM and LPRF-CM (Figure 2a). While 1283 proteins were common between BMSC-CM and LPRF-CM, 874 and 137 proteins were exclusive to BMSC-CM and LPRF-CM, respectively (Figure 2b), revealing a weak intergroup correlation (Pearson R ≥ 0.4) (Figure 2c).

Next, we performed GSEA of the common proteins to identify categories of “over-represented” proteins [37]. Based on the hallmark database, 26 of 35 gene sets were enriched in BMSC-CM. The most highly enriched categories (FDR < 25%) in BMSC-CM included “Epithelial Mesenchymal Transition (EMT)” and “Myc targets V1” (Figure 2d). EMT is the process by which epithelial cells undergo cytoskeleton rearrangement and acquire mesenchymal features resulting in enhanced motility, resistance to apoptosis, and ECM production [38], while MYC (c-Myc), a downstream target of Wnt signaling, regulates stem cell maintenance [39]. In contrast, only 9 of 35 gene sets were enriched in LPRF-CM. Among the six enriched gene sets in LPRF-CM at FDR < 25% were “Heme Metabolism” (possibly due to contamination from erythrocytes), “PI3K AKT mTOR”, “Reactive oxygen species (ROS)”, and “Complement” pathways (Figure 2e). Phosphatidylinositide 3 kinases (PI3Ks) and their downstream mediators AKT and mammalian target of rapamycin (mTOR) are implicated in cell proliferation, survival, and differentiation [40], while ROS and complement proteins regulate immune modulation during normal wound healing [41].

### 2.3. Analysis of DEPs in BMSC-CM and LPRF-CM

**Figure 3:**
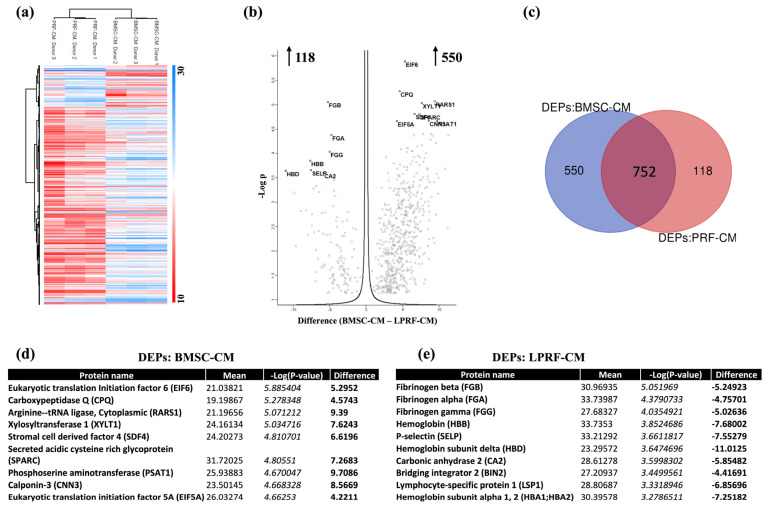
Analysis of differentially expressed proteins (DEPs) in BMSC-CM and LPRF-CM. (**a**) Hierarchical clustering analysis of the DEPs between BMSC-CM and LPRF-CM; lower and higher values are represented in red and blue, respectively. (**b**) Volcano plot showing statistical significance of DEPs (adjusted *p*-value) versus magnitude of expression change (log2 fold change); numbers indicate the upregulated proteins in BMSC-CM (550) and LPRF-CM (118). (**c**) Venn diagram illustrating the number of DEPs for each CM group. Tables listing the top 10 upregulated genes in BMSC-CM (**d**) and LPRF-CM (**e**), ranked by increasing fold change in expression (bold values) including: protein name, the average of the normalized counts taken over all samples (mean), fold change in gene expression (difference) using permutation-based correction for multiple hypothesis testing (FDR = 0.05).

A total of 668 DEPs were identified between the groups; 550 proteins were increased in BMSC-CM and 118 proteins were increased in LPRF-CM (Figure 3a–e). With regards to wound healing/regeneration, several proteins representing GFs, angiogenesis, ECM, and inflammation were increased in BMSC-CM (Table 1; Supplementary File S2). In comparison, relatively fewer proteins related to these processes were increased in LPRF-CM. Immune-related proteins, especially of the complement system, immunoglobulins, and platelet-related proteins were also increased in LPRF-CM. Several non-differentially (or similarly) expressed proteins (*p* > 0.05) related to GFs, angiogenesis-, ECM-, and inflammation-related proteins were also identified in BMSC-CM and LPRF-CM (Table 1). Some GFs were detected exclusively in BMSC-CM or LPRF-CM; notably, several bone- and neural-related proteins were exclusively detected in BMSC-CM (Table 1).

**Figure 4:**
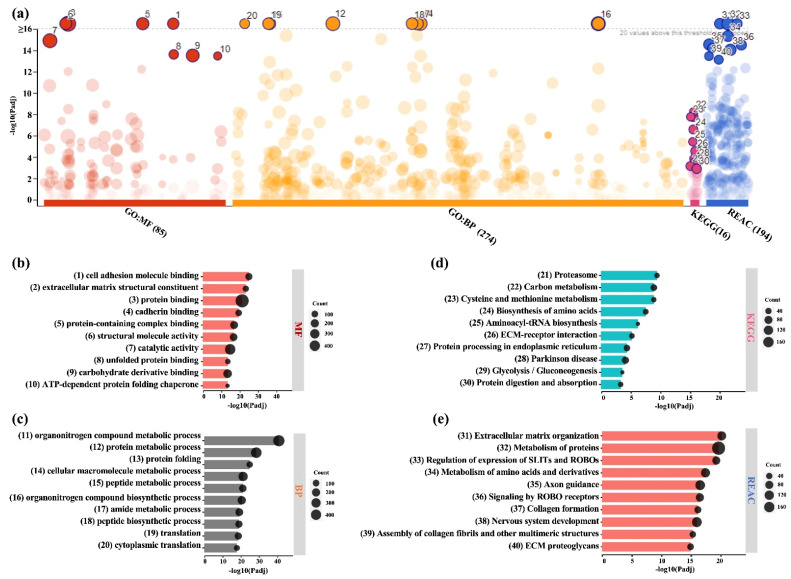
Functional enrichment analysis of DEPs in BMSC-CM. (**a**) Results of enrichment analysis presented in the form of a Manhattan plot, where the *X*-axis shows the functional terms grouped by the color code of source database used and the *Y*-axis shows the enrichment adjusted *p*-values in negative decimal logarithm scale. Dots in the graph indicate all enriched terms meeting the significance criterion of *p* < 0.05, while highlighted dots represent terms filtered by the criterion of top 10 terms. The graphs (**b**–**e**) show the detailed results of the enriched terms highlighted in the Manhattan plot along with the statistical significance (*p*-value) and the number of DEPs belonging to the enriched term (placed next to the bar), according to gene ontology (GO) (**b**) molecular function (MF), (**c**) biological processes (BPs), (**d**) KEGG, and (**e**) REACTOME.

Functional profiling of DEPs in BMSC-CM revealed enrichment of GO categories according to MF (85 terms), BP (274), KEGG (16), and REACTOME databases (196) (Figure 4a). Several of the top 10 enriched MF (“cell adhesion molecule binding”, “extracellular matrix structural constituent”, “protein binding”) and BP terms (“organonitrogen compound metabolic process”, “protein metabolic process”, cellular macromolecule metabolic process”) were related to wound healing (Figure 4b,c). Other MF and BP terms important for wound healing were also identified (Supplementary File S3). These findings were further corroborated by the KEGG (Figure 4d) and REACTOME analysis (Figure 4e, Supplementary File S3). Furthermore, categories related to neural development and function, such as “axon guidance”, “signaling by ROBO receptors”, and “regulation of gene expression (SLITs and ROBOs)”, were also identified in BMSC-CM.

**Figure 5:**
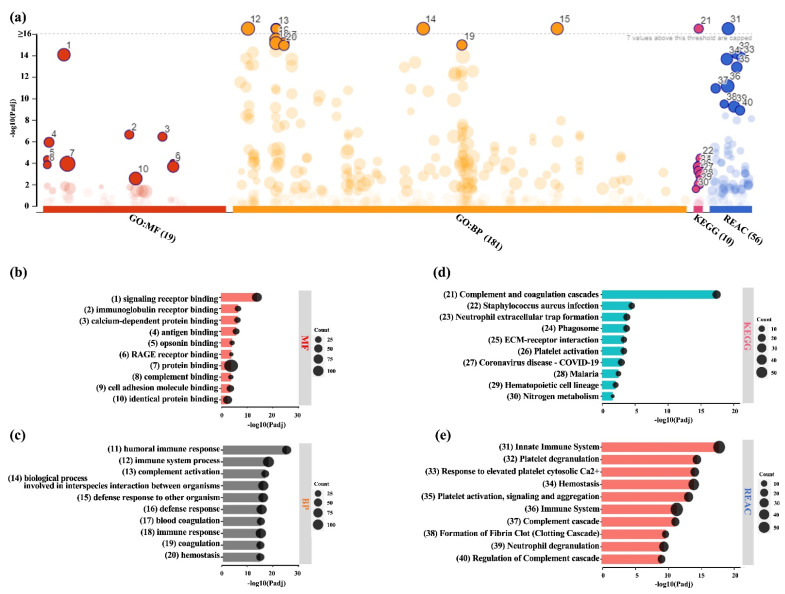
Functional enrichment analysis of DEPs in LPRF-CM (**a**) Results of enrichment analysis presented in the form of a Manhattan plot, where the *X*-axis shows the functional terms grouped by the color code of source database used and the *Y*-axis shows the enrichment adjusted *p*-values in negative decimal logarithm scale. Dots in the graph indicate all enriched terms meeting the significance criterion of *p* < 0.05, while highlighted dots represent terms filtered by the criterion of top 10 terms. The graphs (**b**–**e**) show the detailed results of the enriched terms highlighted in the Manhattan plot along with the statistical significance (*p*-value) and the number of DEPs belonging to the enriched term (placed next to the bar), according to gene ontology (GO) (**b**) molecular function (MF), (**c**) biological processes (BPs), (**d**) KEGG, and (**e**) REACTOME (REAC).

Functional profiling of DEPs in LPRF-CM also revealed significant enrichment according to GO MF (19 terms), BP (181), KEGG (10) and REACTOME (56) databases (Figure 5a). Among the top enriched MF terms were binding (“signaling receptor binding”, “protein binding”, “calcium-dependent protein binding”, “cell adhesion molecule binding”) and immune functions (“antigen binding”, “opsonin binding”, “complement binding”) (Figure 5b). Among the top enriched BP terms were “humoral immune response”, “immune system process”, “complement activation”, “immune response”, and “coagulation/hemostasis” (Figure 5c). Other MF and BP terms important for wound healing were also identified (Supplementary File S3). These findings were further corroborated by the KEGG (Figure 5d) and REACTOME analysis (Figure 5e, Supplementary File S3), especially with regards to immune system-related functions/processes.

Additionally, several GO MF (“protein binding”, “cell adhesion molecule binding”, “integrin binding”, “calcium ion binding”, “glycosaminoglycan binding”, “growth factor binding”) and BP terms (“wound healing”, “response to wounding”, “response to stress/stimulus”, “cell adhesion”, “cell migration”, “cellular response to chemical stimulus”) relevant for wound healing were enriched in both BMSC-CM and LPRF-CM (Supplementary File S3).

### 2.4. Multiplex Immunoassay

**Figure 6:**
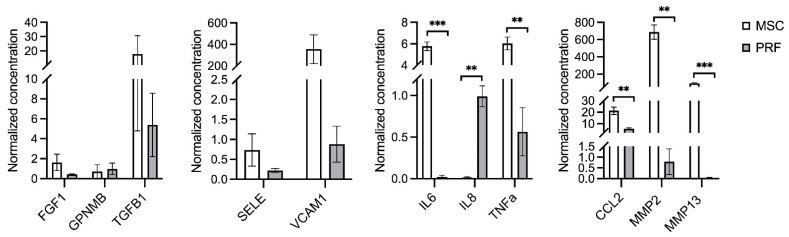
Multiplex immunoassay. Normalized concentrations [cytokine (pg)/total protein (μg)] of cytokines detected in BMSC-CM (MSC) and LPRF-CM (PRF) using a human bone metabolism array (Supplementary Table S1). Data represent means ± standard error of 2–3 independent donors (4 technical replicates per donor). ** *p* < 0.01, *** *p* < 0.001.

A multiplex immunoassay was performed to validate the LC-MS/MS results. Donor-related variations within each CM group were reflected by the intragroup standard deviations (Figure 6). Among the classical GFs, fibroblast growth factor-1 (FGF1), transforming growth factor-beta-1 (TGFβ1), and transmembrane glycoprotein NMB/osteoactivin (GPNMB/OA) were detected in both BMSC-CM and LPRF-CM (*p* > 0.05 for all); FGF2 was detected in BMSC-CM but not in LPRF-CM. Angiogenesis-related E-selectin (SELE) and vascular cell adhesion molecule-1 (VCAM1) were also detected in both groups, with a trend for higher concentrations in BMSC-CM (*p* > 0.05 for both). Inflammatory cytokines interleukin-6 (IL6) and tumor necrosis factor-alpha (TNFA) were increased in BMSC-CM, while the chemokine IL8, also known as CXC-motif chemokine ligand-8 (CXCL8), was increased in LPRF-CM (*p* < 0.01 for all). ECM remodeling-related matrix metalloproteinase-2 (MMP2), -13 (MMP13), and monocyte chemotactic protein-1 (MCP1/CCL2) were increased in BMSC-CM (*p* < 0.01 for all); MMP9 was detected in LPRF-CM but not in BMSC-CM. Similar trends in protein detection were observed in the multiplex and LC-MS/MS analyses.

## 3. Discussion

The secretome of BMSCs is emerging as a promising alternative to cell therapy for tissue regeneration. Indeed, the secretomes of MSCs from different tissues have been extensively characterized, in terms of their contents, using proteomic methods [32,42,43,44]. However, to our knowledge, no previous studies have compared MSC secretomes to a clinical reference of GF-based therapy, such as APCs. Moreover, detailed proteomic characterizations of APCs are limited [16,18,45]. The objective of the present study was to compare the secretome of BMSC to that of L-PRF—an established GF-based therapy—in the context of wound healing and regeneration.

Wound healing is a dynamic and well-orchestrated process consisting of four continuous, temporally overlapping phases. The first phase of hemostasis begins immediately following injury and is characterized by increased platelet activity and fibrin clot formation [5]. Secretion of various bioactive molecules by the platelets (GFs, cytokines, chemokines) results in the infiltration (chemotaxis) of inflammatory and immune cells, such as neutrophils, macrophages, and lymphocytes. A phenotypic “switch” from proinflammatory to anti-inflammatory by macrophages facilitates a transition to the proliferative phase, characterized by mesenchymal cell activity (migration, proliferation, differentiation), angiogenesis, and formation of ECM components—collagens, proteoglycans, etc. Finally, the wound enters a maturation or remodeling phase where the deposited ECM components are reorganized to strengthen and restore the original tissue form [6].

APCs have long been used to enhance wound healing, based on the vital role of platelets in the early stages of the process [9]. L-PRF, a so-called “second generation” APC, has gained significant attention in recent years. The clinical rationale for L-PRF is based on the release of GFs from platelets entrapped in the fibrin matrix [46,47]. More comprehensive proteomic characterizations of the L-PRF “releasate”, i.e., proteins released in culture media with [48] or without manipulation of the fibrin matrix [18], and “lysate”, i.e., proteins extracted via manual disruption of the fibrin matrix [16], have revealed over 700 proteins representing not only GFs, but also various cytokine-, ECM-, and immune-related proteins. These data further support the clinical application of L-PRF in periodontal and bone regeneration [21,23,49,50] and render it as a standard to which newer GF-based strategies, such as MSC secretomes, may be compared.

In the first part of the analysis, we compared the global profiles of BMSC-CM and LPRF-CM. Despite their considerably different origin, BMSC-CM and LPRF-CM showed remarkable similarities in their proteomic profiles. Over 1200 common proteins representing key BP (cellular organization, protein metabolism, etc.) and MF (cell adhesion, protein binding, etc.) involved in wound healing were identified. These proteins included several classical GFs of the TGFβ, bone morphogenetic protein (BMP), vascular endothelial growth factor (VEGF), hepatocyte growth factor (HGF), platelet-derived growth factor (PDGF), stromal cell-derived factor (SDF), and insulin-like growth factor (IGF) family, along with key mediators of angiogenesis and inflammation, all of which are associated with early (inflammation) or intermediate stages (proliferation) of wound healing [51,52]. Moreover, several proteins associated with ECM organization and remodeling, i.e., later stages of healing, were also identified in both BMSC-CM and LPRF-CM.

To reveal the direction of increased/decreased detection of the shared proteins, we identified the DEPs in the two groups; notably, BMSC-CM contained 550 DEPs compared to 118 DEPs in LPRF-CM. Among the top DEPs in BMSC-CM were several ECM-related proteins, while the top DEPs in LPRF-CM mainly represented coagulation, platelet function, and inflammation/immune function. This is consistent with the tissue source of the different CMs, i.e., bone (marrow) and blood (platelets). Moreover, specific proteins known to play a key role in wound healing and regeneration were identified among the DEPs; more healing-related proteins were increased in BMSC-CM compared to LPRF-CM. With regards to bone and periodontal tissues, many of the relevant proteins previously identified in BMSC-CM [53] were also detected in the present study. Among the proteins upregulated in BMSC-CM were classical GFs such TGFβ, BMP, and IGF family proteins, which are known mediators of wound healing [54]. Others [55,56] have reported the presence of several proinflammatory and anti-inflammatory cytokines, which are also relevant for bone regeneration. Moreover, regarding proteins related to angiogenesis, which is a critical step in the healing cascade, both BMSC-CM (VEGFC, VCAM1) and LPRF-CM revealed enriched proteins (von Willebrand factor (VWF), platelet and endothelial cell adhesion molecule-1 (PECAM1)). Indeed, the angiogenic properties of both BMSC-CM and LPRF-CM have previously been demonstrated in vivo [57,58]. Thus, the present data complement the existing evidence regarding the GF contents of MSC secretomes.

In addition to GFs and soluble proteins, several proteins related to EVs were identified herein. Both MSCs and platelets are known to release EVs, i.e., nano-sized membrane encapsulated-bodies (30 nm^−1^ μm), to mediate their paracrine functions. Based on the “classical” EV markers [59], over 40 proteins (CD9, HSPA8, HSP90AA1, ANXA11, ADAM10, etc.) were identified in BMSC-CM and LPRF-CM; of these, 35 proteins were increased in BMSC-CM (CD81, ENO1, HSPA5, HSP90AB1, etc.). MSC-derived EVs have recently gained significant attention in the tissue engineering literature due to their high regenerative efficacy and promising clinical potential [4,60]. Although less researched than MSC-EVs, platelet/PRP-derived EVs have also been demonstrated to improve outcomes in preclinical models of chronic wounds, vascular diseases, bone disorders, etc. [61].

A key finding of the present analysis was the remarkable enrichment of ECM-related proteins in BMSC-CM, which is of particular relevance to periodontal and bone regeneration. Specifically, several proteins related to the organic fraction of bone/periodontal ECM were increased in BMSC-CM (Table 1). This was further confirmed by the functional profiling analysis, which revealed enrichment of processes related to ECM organization and tissue morphogenesis (“biosynthetic process”, “developmental process”, “anatomical structure development”, “multicellular organism development”). The ECM is critical for optimal tissue function, homeostasis, and repair. Moreover, the ECM modulates cell proliferation, adhesion, migration, and differentiation [62]. In bone, the organic ECM (~20–30%) is primarily composed of collagens (mainly of type I (90% of organic ECM), III, and V) and non-collagenous proteins (NCP) [62]. We identified several collagens in both BMSC-CM and LPRF-CM, although bone-specific COL1A1, COL1A2, COL3A1, and COL5A1 were increased in BMSC-CM. Indeed, collagen types I, III, and V also predominantly constitute the periodontal ligament (PDL) [63]. Several NCP, including proteoglycans, Gla-proteins, glycoproteins, SIBLING-proteins, and enzymes, were increased or exclusively identified in BMSC-CM (Table 1). Moreover, several ECM remodeling-related proteins were increased in BMSC-CM. Among these were MMPs along with their inhibitors (TIMPs), which are responsible for ECM degradation [64], and CCL2 [65] and OPG [66], which are inducers and inhibitors, respectively, of osteoclastogenesis and bone resorption. With regards to bone formation, key proteins involved in the TGFβ/BMP and Wnt signaling pathways, which play important roles in bone hemostasis/regeneration [67,68], were increased in BMSC-CM. Several of these ECM proteins are also known to be expressed in the PDL and cementum [69]. Indeed, the efficacy of MSC-CM for PDL/bone regeneration has been demonstrated in preclinical studies [34,35,36]. In context, we have recently demonstrated the efficacy of BMSC-CM in rat calvaria defects, where a trend for greater new bone formation was observed in BMSC-CM-treated vs. allogeneic BMSC-treated defects [70].

In comparison to BMSC-CM, the top enriched BPs in LPRF-CM were related to immune response, coagulation, wound healing, and platelet function. Another important finding of the present study was the remarkable upregulation of immune-related processes in LPRF-CM, including “humoral/innate/adaptive immune response”, “complement activation”, “antimicrobial humoral response”, etc., as confirmed by the functional profiling analysis. Indeed, immune modulation is reported to be a dominant mechanism of MSC activity, with MSCs even being labeled as “immune evasive” due to their ability to suppress/evade host immune responses [30,71]. Platelet derivatives are also known to modulate the functions of immune cells, particularly macrophages [72]. Macrophages play an important role during the early stages of wound healing and their “phenotype switch”, from proinflammatory (M1) to anti-inflammatory (M2), is associated with the transition from an inflammatory to proliferative/reparative phase of wound healing [73]. We [74] and others [75] recently demonstrated the anti-inflammatory effects of PRF lysates and releasates (secretome) on macrophages; polarization towards an M2 phenotype was demonstrated via upregulation of M2-associated genes. Consistently, M2-associated proteins such as ARG1 and CD36 were increased in LPRF-CM in the present study. In context, some immune modulatory proteins, such as galectin-1 (LGALS1) and mannose receptor C type-2 (MRC2), which promote M2 polarization [76], were also increased in BMSC-CM. 

Some limitations of the present study must be acknowledged. Despite our systematic and comprehensive bioinformatic approach, the number of included BMSC and L-PRF donors (*n* = 3 each) was relatively small. Although similar sample sizes are commonly reported in the literature [43,77,78,79], inclusion of additional donors may have provided a clearer picture of donor-related variations in the proteome of each group. Moreover, we analyzed only the secretome of L-PRF, which may not accurately represent the clinical product that includes the fibrin matrix and entrapped cells. In context, our group has previously reported the proteomic analysis of L-PRF lysates [16], while others have characterized the L-PRF secretome [18]. The results herein complement these data with the identification of additional, previously unreported proteins in L-PRF.

## 4. Materials and Methods

### 4.1. Cell Culture and Preparation of BMSC-CM

Details of BMSC isolation and BMSC-CM preparation have been described elsewhere [70,80,80,80,80,80,80,80]. All methods were performed in accordance with the relevant guidelines and regulations. Briefly, following ethical approval (Regional Committees for Medical Research Ethics in Norway, 2013-1248/REK-sør-øst and 2016-1266/REK-nord) and parental informed consent, BMSCs were obtained from bone marrow specimens of systemically healthy donors under standard culture conditions [80]. BMSC-CM was produced from passage 1 (p1) and p2 BMSC (*n* = 3 donors) after 48 h of supplement-free culture [70]. After 48 h, the supernatant media (BMSC-CM) were collected for each donor. The BMSC-CM was centrifuged (4000× *g*, 10 min) to remove any debris, aliquoted, and stored at −80 °C until further use. Additional details of cell culture are provided in the Supplementary Methods.

### 4.2. Preparation of L-PRF and LPRF-CM

L-PRF was prepared according to published protocols [17]. Following local approval (Haukeland University Hospital Bloodbank, Bergen, Norway; AIT-69993) and informed consent, whole blood samples were obtained from healthy volunteer donors (2 females and 1 male; 23–46 years). Three 10 mL glass tubes (A-PRF tubes, Process for PRF, Nice, France) of whole blood were collected per donor via venipuncture and immediately centrifuged (Intra-Spin, BioHorizons, Birmingham, AL, USA) using the recommended settings, i.e., 408× *g* (RCF_clot_) and 653× *g* (RCF_max_) for 12 min at RT [17]. The resulting fibrin clots were gently compressed using the Xpression kit (BioHorizons) for 5 min under gravity pressure to produce the L-PRF membranes. The membranes (*n* = 3 membranes per donor) were each placed in 5 mL supplement-free DMEM under standard incubation with intermittent shaking for 4 h to remove most of the dead cells and plasma proteins. The membranes were washed 3 times with PBS (Invitrogen, Waltham, MA, USA), placed in 6-well plates and cultured in supplement-free DMEM for 72 h [17,18]. After 72 h, the supernatant media (LPRF-CM) from the 3 membranes were collected and pooled for each donor. The LPRF-CM was centrifuged (4000× *g*, 10 min) to remove any debris, aliquoted, and stored at −80 °C until further use.

### 4.3. CM Ultrafiltration

BMSC-CM and LPRF-CM were concentrated using Amicon Ultra-15 3 kDa centrifugal filter devices (Merck Millipore, Billerica, MA, USA) following the manufacturer’s protocol. Briefly, after PBS equilibration, 15 mL of each CM was centrifuged in the Ultra-15 tubes at 4000× *g* for 30 min at 4 °C, followed by buffer exchange with PBS and re-centrifugation at 4000× *g* for 30 min at 4 °C. The corresponding concentrated media (~30-fold) from the individual donors were used for proteomic analysis.

### 4.4. Liquid Chromatography with Tandem Mass Spectrometry (LC-MS/MS)

BMSC-CM and LPRF-CM (*n* = 3 donors each) were analyzed using LC-MS/MS via label-free quantitation (LFQ), as previously described [81]. Briefly, total protein concentration of each sample was measured using a bicinchoninic acid assay (Pierce BCA Kit, Thermo Fisher, Waltham, MA, USA) and 10 μg protein was processed to obtain tryptic peptides. About 0.5 μg protein, as tryptic peptides dissolved in 2% acetonitrile and 0.5% formic acid, was injected into an Ultimate 3000 RSLC system connected online to an Exploris 480 mass spectrometer equipped with EASY-spray nano-electrospray ion source (all from Thermo Scientific, Sunnyvale, CA, USA). Additional details of LC-MS/MS are reported in the Supplementary Methods. The mass spectrometry proteomics data have been deposited to the ProteomeXchange Consortium via the PRIDE partner repository (https://www.ebi.ac.uk/pride/ accessed on 18 April 2023) with the dataset identifier PXD041617 and 10.6019/PXD041617.

### 4.5. Bioinformatic Analysis

LC-MS/MS raw files were searched using Proteome Discoverer software (version 2.5.0.400; Thermo Scientific) and data were analyzed using Perseus software (version 2.3.0.1) [82]. Distributions of proteins in each CM group were determined. Gene ontology (GO) categories based on biological process (BP) and molecular function (MF) were identified using the GO Resource version 17.0 (via PANTHER) [83]. Gene set enrichment analysis (GSEA) of the shared proteins was performed using the GSEA software version 4.3.2 [37], based on human hallmark gene sets (h.all.v2023.1.Hs.symbols.gmt) from the Human MSigDB database. Differentially expressed proteins (DEPs) in BMSC-CM and LPRF-CM were identified using a two-sided Student’s *t*-test in combination with a permutation-based correction for multiple hypothesis testing (FDR = 0.05) using Perseus software. Functional profiling of DEPs in each group was performed using the g:Profiler software (version e108_eg55_p17_0254fbf) [84] based on the human genome and the GO-MF, GO-BP, Kyoto Encyclopedia of Genes and Genomes (KEGG), and REACTOME databases. Additional details of bioinformatic analysis are reported in the Supplementary Methods.

### 4.6. Multiplex Immunoassay

For validation of LC-MS/MS results, a quantibody human bone metabolism array Q1 (RayBiotech Inc., Norcross, GA, USA) (Supplementary Table S1) was performed as previously described [70]. Array hybridization was performed using standard cytokines and samples, i.e., BMSC-CM and LPRF-CM (*n* = 3 donors each; 0.15–0.5 mg/mL of total protein). Array scanning and data extraction were performed as previously described [70]. Protein concentrations were calculated based on linear standard curves and normalized to the corresponding total protein levels.

### 4.7. Statistical Analysis

Identification of DEPs was performed using a two-sided Student’s *t*-test with Fisher’s correction in Perseus software. All other statistical analyses were performed using the Prism 9 software (GraphPad Software, San Diego, CA, USA). Linear data are presented as means (± SD), unless specified. Normality testing was performed using the Shapiro–Wilk test and independent samples *t*-tests with a 0.05 significance level were applied.

## 5. Conclusions

The secretomes of BMSCs and L-PRF revealed several common proteins related to various stages of wound healing, i.e., inflammation, proliferation/angiogenesis, ECM formation, and remodeling. BMSC-CM revealed considerably more DEPs compared to LPRF-CM, including several GFs, chemokines, and ECM molecules. In the context of bone healing, BMSC-CM was most enriched for processes/functions related to cellular function, tissue morphogenesis, and ECM organization, particularly the organic phase of bone ECM, while LPRF-CM was most enriched for immune modulation, coagulation, and platelet function. Further well-designed studies are needed to determine the differences in in vivo efficacy of BMSC-CM and LPRF-CM.

## Figures and Tables

**Table 1:** Selected proteins related to wound healing identified in BMSC-CM and/or LPRF-CM.

Category	Common Proteins	Upregulated Proteins		Exclusive Proteins	
		BMSC-CM	LPRF-CM	BMSC-CM	LPRF-CM
GF-related	FGF1 *, TGFB1, LTBP1, MST1/HGFL, MET/HGFR, HGFAC, HGS, CCN5/CTGFL, PDGFRL, PDGFB, PDGFC, PDGFD, IGF2, IGFALS, CLEC11A/SCGF, KITLG/SCF, HDGFL2, EPS15, EPS15L1, MEGF8, ENAM, GPNMB *	TGFBI, TGFB2, LTBP2, LTBP4, BMP1, EGFR, IGFBP2, IGFBP3, IGFBP4, IGFBP7, CCN2/CTGF, IGF2R, HDGF, MYDGF, CSF1/MCSF, SDF1/CXCL12	TGFB1I1, GRB2	TGFBR2, LTBP3, FGF2 *, FGF7, FGFRL1, PDGFRA, IGFBP5, CCN1/IGFBP10, CCN3/IGFBP9, EPS8, PTN/HBNF	EGF, PDGFA
Cell function-related	PA2G4, NECTIN1, NECTIN2, VCL, CCAR1, BCAM, CKAP5, PCNA	CTNNB1, CDH11, TUBB4B, TUBB, TUBA1A, TUBB3, TUBA1C, PA2G4, TUBB1, PLEC, SDF2, SDF4, SERPINF1	PTK2	WNT5A, WNT5B, CCN4/WISP1, CKAP4, GAP43, CADM1, CDH1, CDH4, CDH13, TUBB2B, TUBB6	F11R, CHL1
Angiogen-esis-related	ANG, ANGPT1, ANGPTL4, ANGPTL6, AGT, VASP, ENG, SELE *	VEGFC, VCAM1, EPCR/PROCR, SVEP1	VWF, PECAM1, ECE1	VEGFA, FLT4/VEGFR3, ANGPTL2, EDF1, AAMP	ICAM2, ICAM3, VWA5A
ECM-related	SPP1/OPN, CLEC3B, ECM1, FN1, FBLN1, FBLN5, FAM20C/DMP4, THBS1, GC, HABP2, CRTAP, EFEMP1, EFEMP2, MXRA8, CHSY3	SPARC/ON, SMOC1, POSTN/OSF2, OGN, BGN, DCN, THBS2, THBS5, MMP1, MMP2, MMP3, TIMP1, TIMP2, TIMP3, CCBE1, COLGALT1, ACTL6A, HAPLN1, ARPC1B, COL1A1, COL1A2, COL3A1, COL4A1, COL4A2, COL5A1, COL5A2, COL6A1, COL6A2, COL6A3, COL8A2, COL11A1, COL12A1, COL14A1, LAMA4, LAMB1, LAMB2, LAMC1, LAMC2, VIM, PCOLCE, MXRA5, PLOD1, PLOD2, CEMIP, MYH10	MMP8, MMP9, CRTAC1	ALPL, OMD, MMP13, MMP14, ARPC1A, HAPLN3, MXRA7, MYH2, COL5A3, COL7A1, COL8A1, COL16A1, CTHRC1, SAFB, SAFB2	ECM2
Inflamma-tion/immune-related	MIF, CCL5/RANTES, CCL18/MIP4, MARCO, CTSD, LGALSL, LGALS3	CCL2/MCP1, TNFA *, TNFAIP6, IL1RAP, ILF2, TNFRSF11B/OPG, OSTF1, CTSB, CTSZ, CTSL, PTGR1, LGALS1, LGALS3BP, MRC2	CXCL8, IL6ST, PRG2, ARG1, CD36	IL6, LIF, TNFRSF1A, EDA2R, IKBKG, CTSF, CTSO, PTGES3	CD14, CD40/TNFRSF5, CLEC1B, CTSG, CTSS, AIF1, HLA-DRA, TNFRSF14, CXCL5, CLEC2B

“Common Proteins” indicate those identified in both BMSC-CM and LPRF-CM (*p* > 0.05); “Upregulated Proteins” indicate those among the commonly identified proteins upregulated in BMSC-CM or LPRF-CM (*p* < 0.05); “Exclusive Proteins” indicate those identified only in BMSC-CM or LPRF-CM. * Detected only in the multiplex assay. GF, growth factors; ECM, extracellular matrix. Full names of proteins are provided in Supplementary File S2.

## Data Availability

The mass spectrometry proteomics data have been deposited to the ProteomeXchange Consortium via the PRIDE partner repository (https://www.ebi.ac.uk/pride/) with the dataset identifier PXD041617 and 10.6019/PXD041617. Additional data are included in the Supplementary data file and can be made available by the authors upon requests addressed to the corresponding author.

## Supplementary Materials

The following supporting information can be downloaded at: https://www.mdpi.com/article/10.3390/ijms241713057/s1.
