# Supplementary material for: Proteomic Analysis of Mesenchymal Stromal Cells Secretome in Comparison to Leukocyte- and Platelet-Rich Fibrin
Source: Int J Mol Sci. 2023 Aug 22;24(17):13057. doi: 10.3390/ijms241713057 (PMC10487446; doi:10.3390/ijms241713057)
Supplement: Supplementary file 1 [file ijms-24-13057-s001.zip › ijms-2502623-supplementary.pdf]

Supplementary methods

Liquid chromatography with tandem mass spectrometry (LC-MS/MS)

LC run (195 min)

Peptides were separated during a biphasic ACN gradient from two nanoflow UPLC pumps (flow rate of 200 nl/min) on a 50 cm analytical column (PepMap RSLC, 50cm x 75 µm ID EASY-spray column, packed with 2µm C18 beads). Solvent A and B were 0.1% TFA (vol/vol) in water and 100% ACN respectively. The gradient composition was 5%B during trapping (5min) followed by 5-8%B over 1 min, 8–25%B for the next 124min, 25-35%B over 30 min, and 35–85%B over 5min. Elution of very hydrophobic peptides and conditioning of the column were performed during 10 minutes isocratic elution with 85%B and 15 minutes isocratic conditioning with 5%B. Instrument control was through Thermo Scientific SII for Xcalibur 1.6.

High field asymmetric waveform ion mobility spectrometry (FAIMS)

The FAIMS Pro interface performs gas-phase fractionation, enabling preferred accumulation of multiply charged ions to maximize the efficiency of data-dependent acquisition (DDA) routines and increase proteome coverage. Short-ion residence time in the FAIMS Pro interface electrode assembly enables use of multiple CV settings in a single run to increase proteome coverage.

DDA with FAIMS

Peptides eluted from the column were detected in the Exploris 480 Mass Spectrometer with FAIMS enabled using three compensation voltages (CVs), -45V, -65V and -80V respectively, and "Advanced Peak Determination" on. During each CV, the mass spectrometer was operated in the DDA-mode (data-dependent-acquisition) to automatically switch between one full scan MS and MS/MS acquisition. Instrument control was through Orbitrap Exploris 480 Tune 3.1 and Xcalibur 4.4. The cycle time was maintained at 1.2s/CV (-45 and -65) or 0.8s/CV (-80). MS spectra were acquired in the scan range 375-1500 m/z with resolution R = 120 000 at m/z 200, automatic gain control (AGC) target of 3e6 and a maximum injection time (IT) at auto (depending on transient length in the orbitrap). The most intense eluting peptides with charge states 2 to 6 were sequentially isolated to standard target value (AGC, usually 1e5) or a maximum IT of 75 ms in the C-trap, and isolation width maintained at 1.6 m/z (quadrupole isolation), before fragmentation in the HCD (Higher-Energy Collision Dissociation. Fragmentation was performed with a normalized collision energy (NCE) of 30 %, and fragments were detected in the Orbitrap at a resolution of 15 000 at m/z 200, with first mass fixed at m/z 120. One MS/MS spectrum of a precursor mass was allowed before dynamic exclusion for 30s with "exclude isotopes" on. Lock-mass internal calibration was not enabled.

Ionsource parameter

The spray and ion-source parameters were as follows. Ion spray voltage = 2000V, no sheath and auxiliary gas flow, and capillary temperature = 275 °C.

Bioinformatic analysis

Venn diagrams were prepared using an online Venn diagram software (https://bioinformatics.psb.ugent.be/webtools/Venn/). Gene Ontology (GO) categories of Biological Process (BP) and Molecular Function (MF) were determined using the Gene Ontology Resource version 17.0 (via PANTHER) with Fisher's Exact Test and False Discovery Rate (FDR) correction. Differentially expressed proteins (DEPs) between BMSC-CM and LPRF-CM were determined using a two-sided Student's *t* test with a permutation-based correction for multiple hypothesis testing (FDR = 0.05), using Perseus software (version 2.3.0.1). In the Gene Set Enrichment Analysis (GSEA), gene sets with statistical significance, i.e., $p < 0.05$, FDR (q) < 0.25 and normalized enrichment score (NES) > 1, were selected and compared to the human hallmark gene sets (h.all.v2023.1.Hs.symbols.gmt) from the Molecular Signatures Database (MSigDB). Functional profiling of DEPs in each group was performed using the g:Profiler software (version e108_eg55_p17_0254fbf) 40 based on the human genome (Homo sapiens) version: GRCh38.p13, and the GO MF (annotations: BioMart classes: releases/2022-12-04), GO BP (annotations: BioMart classes: releases/2022-12-04), Kyoto encyclopedia of genes and genomes (KEGG FTP Release 2022-12-26) and REACTOME databases (annotations: BioMart classes: 2022-12-28). Analysis was performed for annotated genes only. The g:SCS threshold was chosen as the significance threshold and the user threshold was set to $p < 0.05$. The analysis results were then filtered to display only the top-10 terms and only these are highlighted and listed in the Manhattan plots generated by g:Profiler.

Supplementary tables

Supplementary Table S1: List of cytokines included in the human bone metabolism multiplex array (n=31)

AREG	Amphiregulin
BMP4	Bone morphogenetic protein 4
CCL2	C-C motif chemokine 2
CCL3	CC motif chemokine ligand 3
CDH3	P-cadherin
CDH5	VE-Cadherin
CSF1	Colony stimulating factor 1
CXCL12	Stromal cell-derived factor 1
CXCL8	Interleukin 8
FGF1	Fibroblast growth factor 1
FGF2	Fibroblast growth factor 2
GDF2	Growth differentiation factor 2
GPNMB	Glycoprotein nonmetastatic melanoma protein B (osteoactivin)
ICAM1	Intercellular Adhesion Molecule 1
IGF1	Insulin-like growth factor-I
IL11	Interleukin 11
IL17A	Interleukin 17A
IL1A	Interleukin 1 alpha
IL1B	Interleukin 1 beta
IL6	Interleukin 6
INHBA	Inhibin, beta A
MMP13	Matrix metallopeptidase 13
MMP2	Matrix metallopeptidase 2
MMP9	Matrix metallopeptidase 9
SELE	E-Selectin
SHH	Sonic Hedgehog N-Terminal
TGFB1	Transforming growth factor beta 1
TGFB2	Transforming growth factor beta 2
TNF	Tumor necrosis factor
TNFRSF11A	TNF receptor superfamily member 1A (RANK)
VCAM1	Vascular cell adhesion protein 1
